# Intraoperative Facial Nerve Monitoring During Cochlear Implant Surgery: An Observational Study

**DOI:** 10.1097/MD.0000000000000456

**Published:** 2015-01-30

**Authors:** Hui-Shan Hsieh, Che-Ming Wu, Ming-Ying Zhuo, Chao-Hui Yang, Chung-Feng Hwang

**Affiliations:** From the Department of Otolaryngology (H-SH, M-YZ), Chang Gung Memorial Hospital and Xiamen Medical Center, Xiamen; Department of Otolaryngology (C-MW), Linkou Chang Gung Memorial Hospital and Chang Gang University College of Medicine, Linkou; and Department of Otolaryngology (C-HY, C-FH), Kaohsiung Chang Gung Memorial Hospital and Chang Gung University College of Medicine, Kaohsiung, Taiwan.

## Abstract

Iatrogenic facial nerve injury is one of the most severe complications of cochlear implantation (CI) surgery. Intraoperative facial nerve monitoring (IFNM) is used as an adjunctive modality in a variety of neurotologic surgeries. The purpose of this retrospective study was to assess whether the use of IFNM is associated with postoperative facial nerve injury during CI surgery. The medical charts of 645 patients who underwent CI from 1999 to 2014 were reviewed to identify postoperative facial nerve palsy between those who did and did not receive IFNM. Four patients (3 children and 1 adult) were found to have delayed onset facial nerve weakness. IFNM was used in 273 patients, of whom 2 had postoperative facial nerve weakness (incidence of 0.73%). The incidence of facial nerve weakness was 0.54% (2/372) in the patients who did not receive IFNM. IFNM had no significant effect on postoperative delayed facial palsy (*P* = 1.000). All patients completely recovered within 3 months after surgery. Interestingly, all 4 cases of facial palsy received right CI, which may be because all of the surgeons in this study used their right hand to hold the drill. When right CI surgery is performed by a right-handed surgeon, the shaft of the drill is closer to the inferior angle of the facial recess, and it is easier to place the drilling shaft against the medial boundary (facial nerve) when the facial recess is small. The facial nerve sheaths of another 3 patients were unexpectedly dissected by a diamond burr during the surgery, and the monitor sounded an alarm. None of these 3 patients developed facial palsy postoperatively. This suggests that IFNM could be used as an alarm system for mechanical compression even without current stimulation. Although there appeared to be no relationship between the use of monitoring and delayed facial nerve palsy, IFNM is of great value in the early identification of a dehiscent facial nerve and assisting in the maintenance of its integrity. IFNM can still be used as an additional technique to optimize surgical success.

## INTRODUCTION

Surgical rehabilitation by cochlear implantation (CI) has become the treatment of choice for severely deaf people who did not sufficiently benefit from hearing amplification. The reported overall major complication rate after CI surgery is relatively low at approximately 2.3% to 8%.^1^ Facial nerve paralysis is a rare but devastating complication of CI surgery, with a reported incidence rate ranging from 0.67% to 1.2%.^2–4^

Intraoperative continuous facial nerve monitoring (IFNM) using an electromyograph (EMG) was first established in neurotologic surgery.^5,6^ This technique monitors muscles innervated by the facial nerve at risk during surgery. Iatrogenic trauma to nerves evokes high-frequency bursts of motor unit potentials called neurotonic discharges that are detected by a monitor.^5,7^ This alerts the surgeon and may help to prevent serious or irreversible injury. Routine intraoperative nerve monitoring during thyroid and parathyroid surgery has gained widespread acceptance as an adjunct to the gold standard of visual identification of the recurrent laryngeal nerve.^8^ Many studies have evaluated EMG monitoring for the prevention of iatrogenic facial injury during parotid surgery; however, the results have been conflicting.^9,10^

The routine use of a neuromonitor has not yet been established as a standard procedure in otologic surgery.^11^ The purpose of this retrospective study was to assess whether the use of IFNM using an EMG is associated with postoperative facial nerve injury, and share our experience during CI surgery.

## METHODS

### Patients

After obtaining the approval of the institutional review board (No. 103-48917), a retrospective review of the clinical data of 645 patients who underwent consecutive CI surgeries with or without IFNM between 1999 and 2014 was completed. We used a facial nerve monitor (Nerve Integrity Monitor; Medtronic Xomed, Jacksonville, FL) for IFNM.

**FIGURE 1:**
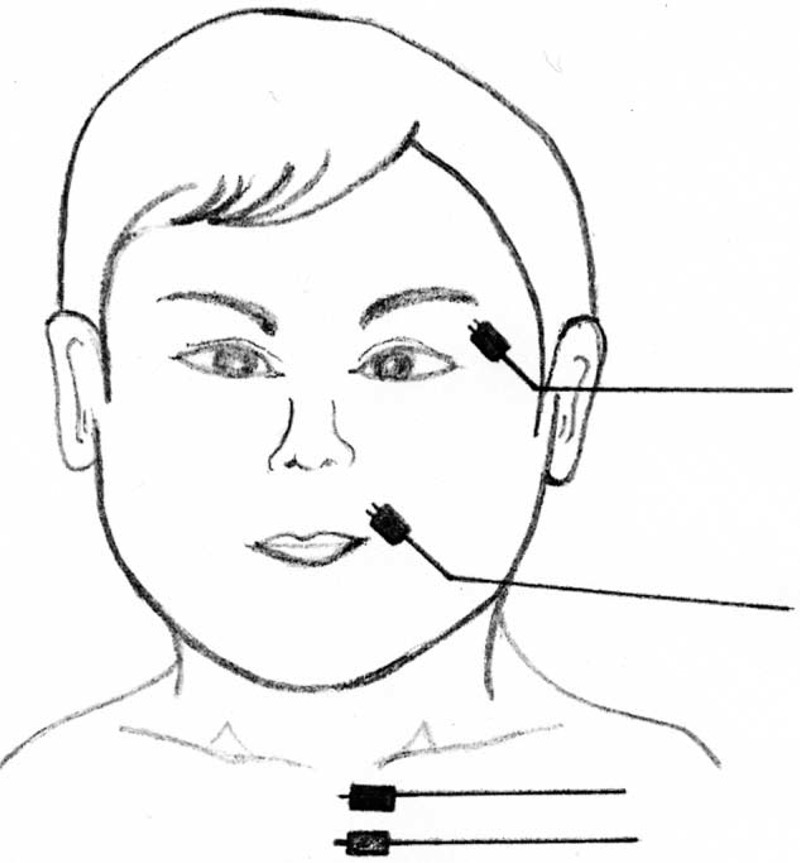
A 2-channel EMG system monitored 2 mimic muscles (musculus orbicularis oculi and musculus orbicularis oris) during the surgery. Two subdermal paired monopolar electrodes were placed 10 and 15 mm distal to the upper eyelid (musculus orbicularis oculi), and 10 and 15 mm cranial to the corner of the mouth (musculus orbicularis oris), respectively. Ground and stimulant return electrodes were also placed into the dermis of the anterior chest. EMG = electromyography.

All surgeries were performed under general anesthesia using an operating microscope in a standard manner. A 2-channel EMG system monitored 2 mimic muscles (musculus orbicularis oculi and musculus orbicularis oris) during the surgery. Two subdermal monopolar paired needle electrodes were placed 10 and 15 mm distal to the upper eyelid (musculus orbicularis oculi), and 10 and 15 mm cranial to the corner of the mouth (musculus orbicularis oris), respectively (Figure 1). A baseline recording was obtained before beginning the operation by tapping the skin of the electrode insertion. Muscle relaxants were not given to exclude any compromise of facial EMG monitoring capability during surgery. The facial nerve was directly stimulated with a monopolar stimulator (constant current pulses, 0.1–3.0 mA, 4 Hz, 100 μsecond), and an event threshold was set at 100 μV. The monitor indicated unexpected facial contractions by sounding an alarm.

**FIGURE 2:**
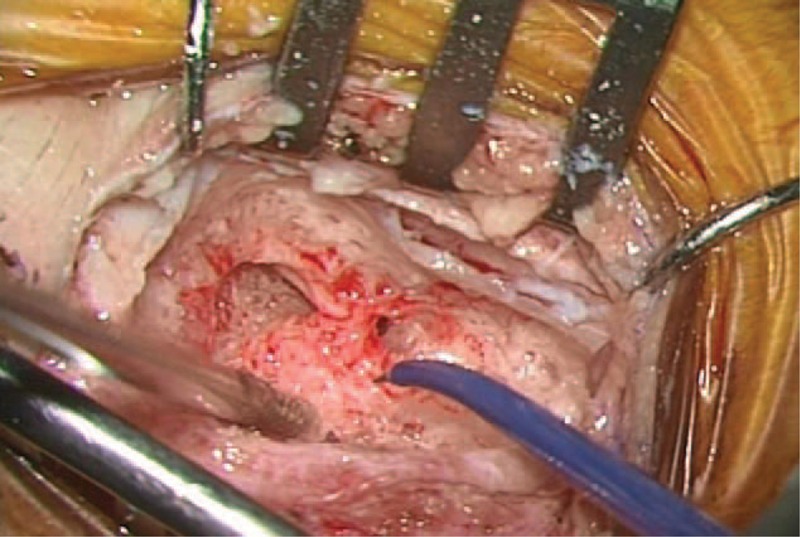
After simple mastoidectomy, the probe tip was used to map out the location of the nerve within the bone, while surgeons though close to the facial nerve.

CI was performed with an inverted J or minimally invasive incision. After simple mastoidectomy, the probe-tip was used to map out the location of the nerve within the bone, while surgeons though close to the facial nerve (Figure 2). Typically, the current level needed to stimulate the horizontal or vertical facial nerve when covered by a 1-mm intact bone was around 1.0 mA.^11,12^ Thus, the initial stimulation current level in our series was set at 1.0 mA. Minimal stimulation thresholds were determined by decreasing the stimulating intensity in decrements of 0.1 mA until recordable EMG responses were obtained. If the probe did not induce the monitor to sound an alarm after the facial recess was skeletonized to visualize the stapes and round window, this indicated that the facial nerve was still covered by thick bone and that it was away from the surgical field. The stimulating intensity was then increased from 1.0 mA to map out the course and position of the facial nerve.

### Clinical Assessment

Facial nerve function was analyzed according to the House–Brackmann (HB) facial nerve grading scale,^13^ preoperatively, at day 1 after surgery, and during the follow-up period. The patients were followed postoperatively until the facial nerve had recovered or permanent weakness was determined. The timing of onset was defined as follows: immediate onset palsy, occurring within 1 hour of surgery; early-onset delayed palsy, occurring between 1 and 48 hours; and late-onset delay palsy occurring beyond 48 hours.^3,14^

### Statistical Analysis

Statistical analysis of the data was performed using SPSS software version 16.0 (SPSS Inc, Chicago, IL) with *t*, χ^2^, and Fisher exact tests as appropriate. Data were presented as means ± standard deviation. Nominal 2-sided *P* values are reported, and the significance level was set at *P* < 0.05.

## RESULTS

### Patients’ Characteristics

Six hundred forty-five patients (299 females and 346 males) were included in the study, with a mean age of 8.3 ± 12.3 years (range 1.0–74.1 years). A total of 645 CI surgeries were performed using the same standardized technique at 3 branches of the same medical center. In total, 273 patients underwent CI surgery using IFNM (IFNM group) and 372 patients underwent without using IFNM (control group) from 1999 to 2014 (Table 1). In the IFNM group, there were more right side CI surgeries than left (*P* = 0.005). The surgeon at the Xiamen branch significantly performed more right CI surgeries and used facial nerve monitoring significantly more than those in the other branches (*P* < 0.001).

**TABLE 1:**
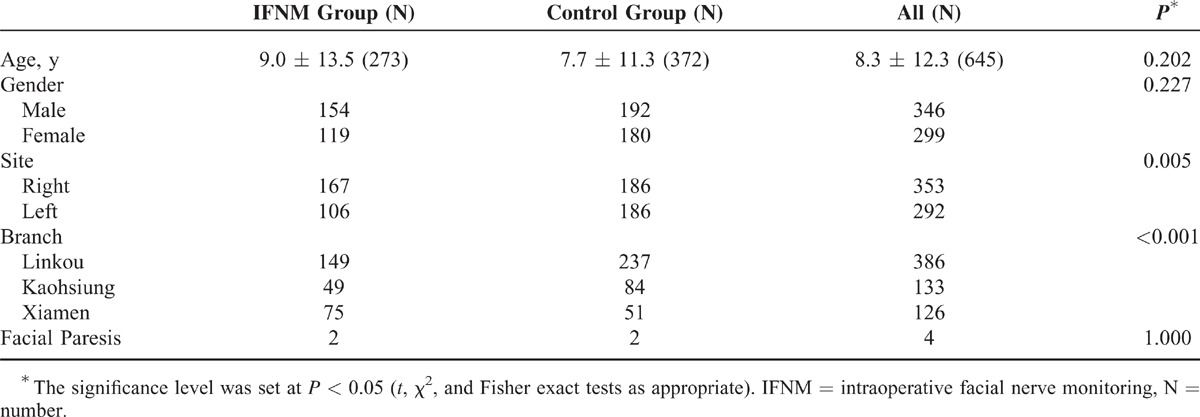
Clinical Characteristics of the 645 Cochlear Implant Patients

Of the 645 patients, 4 patients (3 children and 1 adult) developed delayed onset facial nerve palsy, incidence rate 0.62% (4/645) (Table 2). All of the patients completely recovered within 3 months after surgery. The incidence of transient facial palsy was slightly higher in the IFNM group (2/273, 0.73%) than in the control group (2/372, 0.54%); however, the difference was not statistically significant (*P* = 1.000). IFNM had no significant effect on postoperative nerve facial outcomes. However, in the 2 cases with transient facial palsy where IFNM was employed, neural integrity testing was intact, and the monitor did not sound an alarm during the mastoidectomy or cochleostomy. Three minor complications with IFNM were noted postoperatively, including 2 patients with skin bruises at the needle insertion site and 1 patient with facial twitching for 2 days.

**TABLE 2:**
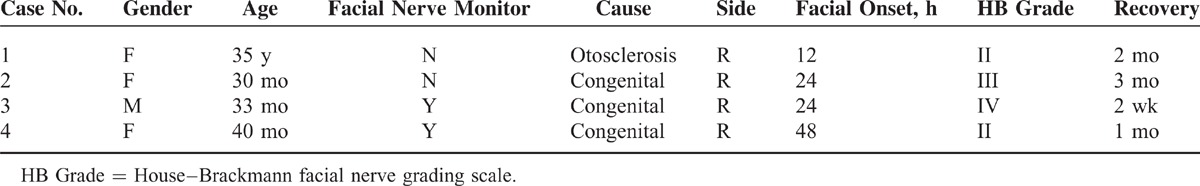
Characteristics of 4 Patients With Facial Nerve Palsy After Cochlear Implantation

### Illustrative Cases

In the control group, 2 patients were found to have postoperative facial nerve palsy. A 35-year-old woman (case 1) who had bilateral profound sensorineural hearing loss because of otosclerosis was evaluated for CI after she no longer benefited from amplification. Her facial nerve was intact and no difficulties were encountered during the surgery. Twelve hours after surgery, she developed right side facial palsy of HB grade II. Her facial nerve function became normal 2 months later. The other case was a 2.5-year-old girl (case 2) with bilateral congenital sensorineural hearing loss who underwent right CI. Her facial nerve was not exposed during surgery and full electrode insertion was achieved. The next day, she had HB grade III facial nerve function, which recovered completely within 3 months. Neither of the patients received steroids or antiviral drugs.

In the IFNM group, 2 pediatric patients (cases 3 and 4) with bilateral congenital sensorineural hearing loss underwent right CI and experienced delayed facial nerve paralysis. They both had a normal facial nerve position (with probe-tip identifications) and their facial nerves were not exposed during surgery. Full insertion of the implants was achieved; however, they developed postoperative HB grades IV and II facial paralysis at 24 and 48 hours, respectively. A tapered dosage cortisone was used in both the patients, one of whom fully recovery in 2 weeks and the other in 1 month.

The facial nerve sheaths of 3 patients were exposed during surgery; however, no injuries resulted. All of the patients had bilateral profound sensorineural hearing loss and underwent right CI. During the procedure when the surgeon was working within the mastoid, soft tissue was suddenly encountered with the diamond burr and the alarm sounded. Stimulation at 1.0 mA elicited both an alarm from the monitor and a facial contraction. This indicated that although facial nerve sheaths were unexpectedly encountered, injures were prevented. None of these patients had facial paralysis.

## DISCUSSION

CI is a safe and reliable surgical procedure; this study demonstrates that facial nerve paralysis following CI is rare. The incidence of facial palsy in our series was 0.62%, which is similar to that reported by other authors.^2–4^ All of the patients who experienced facial palsy had the early-onset delayed type (ie, none was in the short term) and eventually recovered normal facial function.

Intraoperative factors including mechanical trauma and thermal injury can lead to progressive inflammation, neural edema, and ischemia, which may, in turn, result in immediate or early-onset delayed palsy. Among these possible factors, thermal injury was most likely the cause in our series because all cases had early-onset delayed facial palsy and the nerve sheaths were not exposed during surgery. Thermal injury may occur because of inadequate irrigation or overly vigorously drilling the facial recess. In several previous reports, thermal injury was also reported to occur when the drilling shaft was accidentally placed against the facial nerve when drilling during cochleostomy.^2,15^ Use of copious irrigation can reduce the possibility of heating the burrs, and thus decrease the risk of thermal injury and neural edema.

**FIGURE 3:**
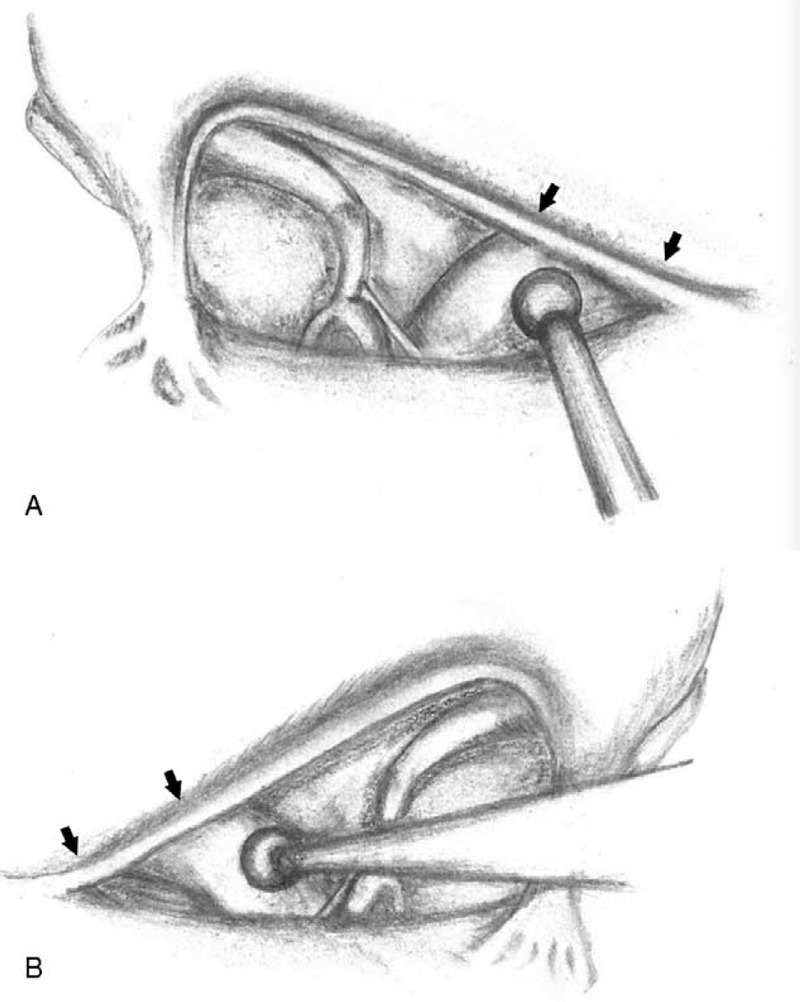
The width of the facial recess was smaller in the inferior angle formed by the chorda tympani nerve (arrow). (A) When right CI surgery was performed by right-handed surgeon, the shaft of the drill was closer to the inferior angle of the facial recess. (B) When left CI surgery was performed by a right-handed surgeon, the drilling shaft was farther from the inferior angle of the facial recess. CI = cochlear implantation.

Interestingly, all the 4 cases with facial palsy received right CI, which may be because all of the surgeons in this study used their right hand to hold the drill. As the facial recess is defined as the aerated extension posterior superior portion of the middle ear cavity medial to the tympanic annulus and chorda tympani and lateral to the fallopian canal (facial nerve), the width of the facial recess is smaller in the inferior angle formed by the chorda tympani nerve (Figure 3). When right CI surgery is performed by a right-handed surgeon, the shaft of the drill will be closer to the inferior angle of the facial recess, and it is easier to place the drilling shaft against the medial boundary (facial nerve) when the facial recess is small. However, during cochleostomy, the surgeon may focus only on how to open the scala tympani. Thermal injury can occur under such circumstances, and this is why we speculate that thermal injury was the major cause of facial nerve palsy in our cases. This relationship between hand positioning of the surgeons and anatomy of the facial recess has never been reported in the literature. Further investigations are required to confirm our hypothesis.

To the best of our knowledge, no study has focused on the effectiveness of IFNM in diminishing the risk of postoperative facial palsy in CI surgery. Only 2 articles have briefly discussed the issue; however, their results were controversial.^2,3^ Fayad et al^3^ reported that there is no relationship between the use of monitoring and delayed facial nerve palsy. However, Thom et al^2^ observed a 4.5-fold increased risk of delayed postoperative facial nerve palsy in cases when IFNM was not used. In this study, the incidence rates of delayed-onset facial palsy in the cases who did and did not receive IFNM were 0.73% and 0.54%, respectively, and the difference was not significant (*P* = 1.000). This means that the use of IFNM may not decrease the risk of thermal injury and early-onset delayed facial palsy. As previously mentioned, we speculate that the cause of early-onset facial nerve paresis in the 2 cases in the IFNM group (Table 2, cases 3 and 4) was most likely due to heat transfer from the drill shaft during the cochleostomy. There was no obvious direct mechanical injury to the facial nerve and the monitor did not sound an alarm during the cochleostomy in either of these cases.

Despite the fact that our results imply a loose association between the use of IFNM and postoperative facial nerve function and thus fail to validate the standardized use of nerve monitoring in CI surgery, we still acknowledge the merit of using an EMG monitoring system as an additional precautionary device to prevent further nerve injuries, especially considering that 3 cases in our series were found to have incidental facial sheath exposure during drilling. IFNM is also useful in training surgeons and enhancing their skills under the guidance of experienced surgeons.^16^ Moreover, the use of IFNM is strongly encouraged in cases with otitis media, mastoiditis, or abnormal inner ear morphology because of the poor visibility and lower predictability of the location of the facial nerve in these patients.

The facial nerve sheaths of 3 patients were unexpectedly dissected by the diamond burr during surgery, and the IFNM sounded an alarm. None of these 3 patients developed facial palsy postoperatively, suggesting that IFNM could be used as an alarm for mechanical compression even without current stimulation. In addition, it is of great value in the early identification of a dehiscent facial nerve and in assisting the maintenance of its integrity.

A previous study reported that 3 patients who received IFNM suffered serious facial skin burns due to defects in the monitoring device in 1999; however, there have not been any similar reports since the facial nerve monitoring apparatus has been improved.^17^ In our experience of using IFNM, we have not encountered major complications. However, 3 patients with minor complications were noted postoperatively in our series. Two of these patients had mild bruises at the needle insertion site, implying that direct insertion into facial vessels should be avoided to prevent severe hematoma. In addition, we suggest that pressure compression should be applied on the insertion site for at least 1 minute after removing the electrical needle.

The other patient with a minor complication developed facial twitching because the suspected location of the horizontal facial nerve was touched twice with an electrical current of only 1.0 mA as a demonstration to an intern. The twitch subsided 2 days later, and no facial weakness was noted thereafter. In theory, it is possible that a strong electrical current may damage the facial nerve. The minimum current needed to stimulate the facial nerve is different, and has been reported to range from 0.75 to 1.0 mA for horizontal or vertical facial nerves.^12^ Hence, the initial stimulation current level was set at 1.0 mA in this study. We do not recommend setting a current higher than 1.0 mA as the initial stimulation intensity because facial nerve sheaths may be exposed during CI surgery, and there is, therefore, a risk of facial nerve injury if the initial stimulating current level is too high.

This retrospective study is limited because of a lack of random control group and because the CI surgeries were not performed by the same doctor. Of note, only 4 cases (0.62%) of a large sample of 645 implanted patients were found to have facial nerve palsy postoperatively. This shows that the incidence of this complication is relatively low, most likely because the facial nerve is in a specific location (ie, inside the facial canal) for most patients and because its direction is predictable, thus making it less likely to be injured during surgery. However, it requires experienced surgeons to make judgments on the direction of the facial nerve and the likelihood of nerve sheath exposure. Therefore, despite the assistance from IFNM, surgeons still need to gain experiences to avoid injury.

## CONCLUSIONS

Although there appears to be no relationship between the use of monitoring and delayed facial nerve palsy, IFNM is of great value in the early identification of dehiscent facial nerves and in assisting the maintenance of its integrity. IFNM can still be used as an additional technique to optimize surgical success.

## Acknowledgment

The authors are grateful to Professor Hsueh-Wen Chang(National Sun Yat-sen University, Department of Biological Science, Taiwan) for his excellent statistical consultation.
